# Online interoperable resources for building hippocampal neuron models via the Hippocampus Hub

**DOI:** 10.3389/fninf.2023.1271059

**Published:** 2023-11-01

**Authors:** Luca Leonardo Bologna, Antonino Tocco, Roberto Smiriglia, Armando Romani, Felix Schürmann, Michele Migliore

**Affiliations:** ^1^Institute of Biophysics, National Research Council, Palermo, Italy; ^2^Blue Brain Project, École Polytechnique Fédérale de Lausanne, Geneva, Switzerland

**Keywords:** hippocampus, research hub, data-driven brain models, online resources, EBRAINS

## Abstract

To build biophysically detailed models of brain cells, circuits, and regions, a data-driven approach is increasingly being adopted. This helps to obtain a simulated activity that reproduces the experimentally recorded neural dynamics as faithfully as possible, and to turn the model into a useful framework for making predictions based on the principles governing the nature of neural cells. In such a context, the access to existing neural models and data outstandingly facilitates the work of computational neuroscientists and fosters its novelty, as the scientific community grows wider and neural models progressively increase in type, size, and number. Nonetheless, even when accessibility is guaranteed, data and models are rarely reused since it is difficult to retrieve, extract and/or understand relevant information and scientists are often required to download and modify individual files, perform neural data analysis, optimize model parameters, and run simulations, on their own and with their own resources. While focusing on the construction of biophysically and morphologically accurate models of hippocampal cells, we have created an online resource, the Build section of the Hippocampus Hub -a scientific portal for research on the hippocampus- that gathers data and models from different online open repositories and allows their collection as the first step of a single cell model building workflow. Interoperability of tools and data is the key feature of the work we are presenting. Through a simple click-and-collect procedure, like filling the shopping cart of an online store, researchers can intuitively select the files of interest (i.e., electrophysiological recordings, neural morphology, and model components), and get started with the construction of a data-driven hippocampal neuron model. Such a workflow importantly includes a model optimization process, which leverages high performance computing resources transparently granted to the users, and a framework for running simulations of the optimized model, both available through the EBRAINS Hodgkin-Huxley Neuron Builder online tool.

## 1. Introduction

The importance of data-driven neural models in the study of the brain is proven by the number of papers that use computational representations of single cells and neural tissues to understand brain mechanisms not yet accessible to the experimentalists and/or make predictions on neural dynamics still unknown (Einevoll et al., 2019).

As for neural modeling and simulation frameworks, computational neuroscientists have witnessed, fostered and taken advantage of a growing interest in the development of software tools for modeling the activity of neural cells at different scales and levels of details (Hines and Carnevale, 1997, 2001; Bower and Beeman, 2007; Gewaltig and Diesmann, 2007; Eppler, 2008; Goodman and Brette, 2008). At the same time, several books and articles have been published so as to help scientists to choose and build their own models (Rieke, 1999; Koch and Segev, 2001; Gerstner and Kistler, 2002; Izhikevich, 2003, 2004; Bower, 2013). Also, scientific repositories/inventories of neural models and platforms/tools for model building, often publicly exposed through web portal or dedicated services (e.g., REST APIs) have been created and are currently maintained. For example, ModelDB (McDougal et al., 2017) collects a large ensemble of curated models that implements, via a variety of modeling languages, such as NEURON (Hines and Carnevale, 1997, 2001; Carnevale and Hines, 2006), NEST (Gewaltig and Diesmann, 2007) and Python, neural mechanisms relative to different cell types, currents, receptors, and transmitters at different scales (e.g., single cells, small and medium size circuits). The OpenSourceBrain (OSB) (Gleeson et al., 2019) allows simulation (as well as visualization, analysis and sharing) of neural models standardized via the PyNN and NeuroML model description languages (Davison, 2008; Gleeson et al., 2010). Also, the NeuroML-DB^1^ that stores over 1,500 models translated to NeuroML2 (Cannon et al., 2014) and provides reciprocal links to ModelDB and OSB, has been introduced (Birgiolas et al., 2023).

Most importantly, a modeling study cannot help but take into consideration the data on which to base the model construction and model validation phases. In the neuroscience field, data are available from several sources: the DANDI Archive, supported by the BRAIN Initiative (Insel et al., 2013; Litvina et al., 2019), gives access to neurophysiology data such as images and electrophysiological and behavioral recordings; the Allen Institute for Brain Science^2^ makes available a plethora of data and tools such as atlases, cell types and datasets, connectivity data and analysis toolkits; the OpenNEURO repository allows to validate and share imaging data such as MRI, PET, MEG, and EEG acquisitions;^3^ the NeuroMorpho curated inventory provides reconstructed morphologies associated with scientific publications and contributed by hundreds of laboratories.^4^ In addition, non-neuroscience specific resources are also available such as Figshare^5^ and Dryad.^6^

Recently, a comprehensive resource has been added to those available to the scientific community: the EBRAINS Knowledge Graph (KG, https://docs.kg.ebrains.eu/), designed and developed in the framework of the Human Brain Project (HBP) (Amunts et al., 2016) and the EBRAINS Research Infrastructure.^7^ This online data and metadata management system provides access to curated data and models as well as tools and services, by exposing all relevant metadata and links (including download and execution urls) for their use. The KG is accompanied by the EBRAINS Model Catalog (MC, https://model-catalog.brainsimulation.eu/) that allows the users to register their own models and have them immediately available (prior to the curation phase needed for the publication in the KG) and also provides –and allows to contribute with– results related to the validation of neural models against electrophysiological data (Sáray et al., 2021). Recently, the EBRAINS ecosystem has been enriched by the publication of the EBRAINS Live Papers portal,^8^ an innovative resource that complements scientific articles with links to related data and models as well the visualization and simulation frameworks needed for their exploration and use (Appukuttan et al., 2022).

Notwithstanding the wide and increasingly rich scenario of online and publicly accessible repositories and services, an instrument that transparently and constantly monitors neuroscience-related online resources, and provides the opportunity to integrate the computational assets they offer to create and optimize biophysically detailed models of individual cells, is still missing. For example, the OSB (now at its v2 release) is a very rich resource that allows to create repositories or link them from external platforms (e.g., GitHub), build workspaces for collaborative research and provides the users with *ad hoc* developed frameworks for integrating and using powerful tools and environments such as the Neurodata Without Borders (NWB) Explorer, NetPyNE and JupyterLab. OpenWorm is an open source project (Szigeti et al., 2014; Sarma et al., 2018) that aims at creating a virtual organism by leveraging standard technology (e.g., the NeuroML language) while contributing to community-oriented initiative such as the above mentioned OSB. The EBRAINS collaborative environment^9^ and tools and services ecosystem for modeling and simulation^10^ offer virtual workspaces, APIs, software packages and interactive frameworks (e.g., JupyterLab) for building, visualizing and analyzing models and simulation results.

However, all these resources do not offer a transparent and automatic search engine able to explore well established remote repositories in quest of model components (for example all the reconstructed and curated morphologies of a specific brain region) or provide HPC facilities to be used in the data-driven model optimization process for free and with no need for any programming step.

To fill this gap, based on the expertise of our group in modeling biophysically detailed individual cells (especially through the NEURON environment) as well as larger scale circuits of the hippocampus (Migliore et al., 2018; Romani et al., 2022; Centofante et al., 2023; Gandolfi et al., 2023) and driven by the neuroscientific community’s need for a tool that saves the users a time-consuming search of online resources as well as manual procedures for model building and optimization, we created the Build section of the Hippocampus Hub (HH).^11^ This resource allows users to monitor the availability of hippocampus related online resources and use them to build their own models. The chosen resources are then seamlessly transferred to and managed by the EBRAINS Hodgkin-Huxley Neuron Builder (HHNB), an online tool for electrophysiological feature extraction, model optimization and simulation, that lifts the burden of software installation and use off the users, through an intuitive and user-friendly web interface (see section *The EBRAINS Hodgkin-Huxley Neuron Builder*). Neural data are available from internal sources (i.e., they are provided by the HH creators, stored in the KG and available in the HH Explore section), digitally reconstructed morphologies are fetched from both the HH Explore section and the NeuroMorpho inventory, while biophysical mechanisms models are retrieved from the ModelDB repository. Additionally, details on the hippocampal circuitry are provided from the Hippocampome portal. All data can be downloaded and/or visualized interactively (in case a graphical representation is available) and the link to the original source, namely the repository from which the data and their relative information are obtained, is provided.

Via the interactive web interface, users can fill a cart with all the components instrumental for building the model they are interested in and transfer the selected resources to the HHNB. The outcome of the entire process, namely the optimized model, can be saved in the MC, and made publicly available to the scientific community.

## 2. Methods

### 2.1. Open hippocampus repositories

The key feature of the HH Build section is its capability to provide the users with an overall view of the resources, related to the computational modeling of the hippocampus, available on remote repositories and to make them available to the users for model building (see sections *Implementation and deployment* and *Usage*). Such resources are those needed for the construction of a NEURON hippocampal cell model in a model building workflow: (1) morphologies, (2) electrophysiological traces, and (3) model components (i.e., digital representations of the biophysical mechanisms governing the behavior of neural cells). For this first release of the HH, we focused our effort on integrating morphologies and model components from two *de facto* standard repositories: the NeuroMorpho inventory,^12^ providing digitally reconstructed neuron morphologies from hundreds of laboratories worldwide (Akram et al., 2018) (currently, the Neurolucida ASCII format *.asc* and the standardized *.swc* format are available) and the ModelDB portal (https://senselab.med.yale.edu/ModelDB/, McDougal et al., 2015, 2017), where all the components of a single model, namely the *.mod* files describing the biophysical mechanisms in the NEURON simulator format, can be selected individually. As for the electrophysiological traces, the actual dataset is exposed in the HH Explore section (currently all the data are in the Axon Binary File, namely *.abf*, format), is available in the KG and is managed and made available through the Blue Brain Nexus ecosystem. These data have been characterized and adopted for model construction in previous modeling work (Migliore et al., 2018). While it is the users’ responsibility to choose the data and model components more suitable for their modeling objectives, the quality of the items exposed through the HH is ensured by the curation process they undergo before publication on the relative respositories.

Finally, we also provide the users with a set of entries describing the circuitry of different hippocampal regions, fetched from the Hippocampome^13^ resource (Wheeler et al., 2015). A thumbnail indicating the position of the cell of interest in its brain region is displayed, and a link to the source web page, where additional details are given, is provided. While this information is not used in the model building workflow, yet we believe it helps the users to get a more comprehensive image of the structure and functioning of the hippocampus.

### 2.2. Implementation and deployment

**Figure 1:**
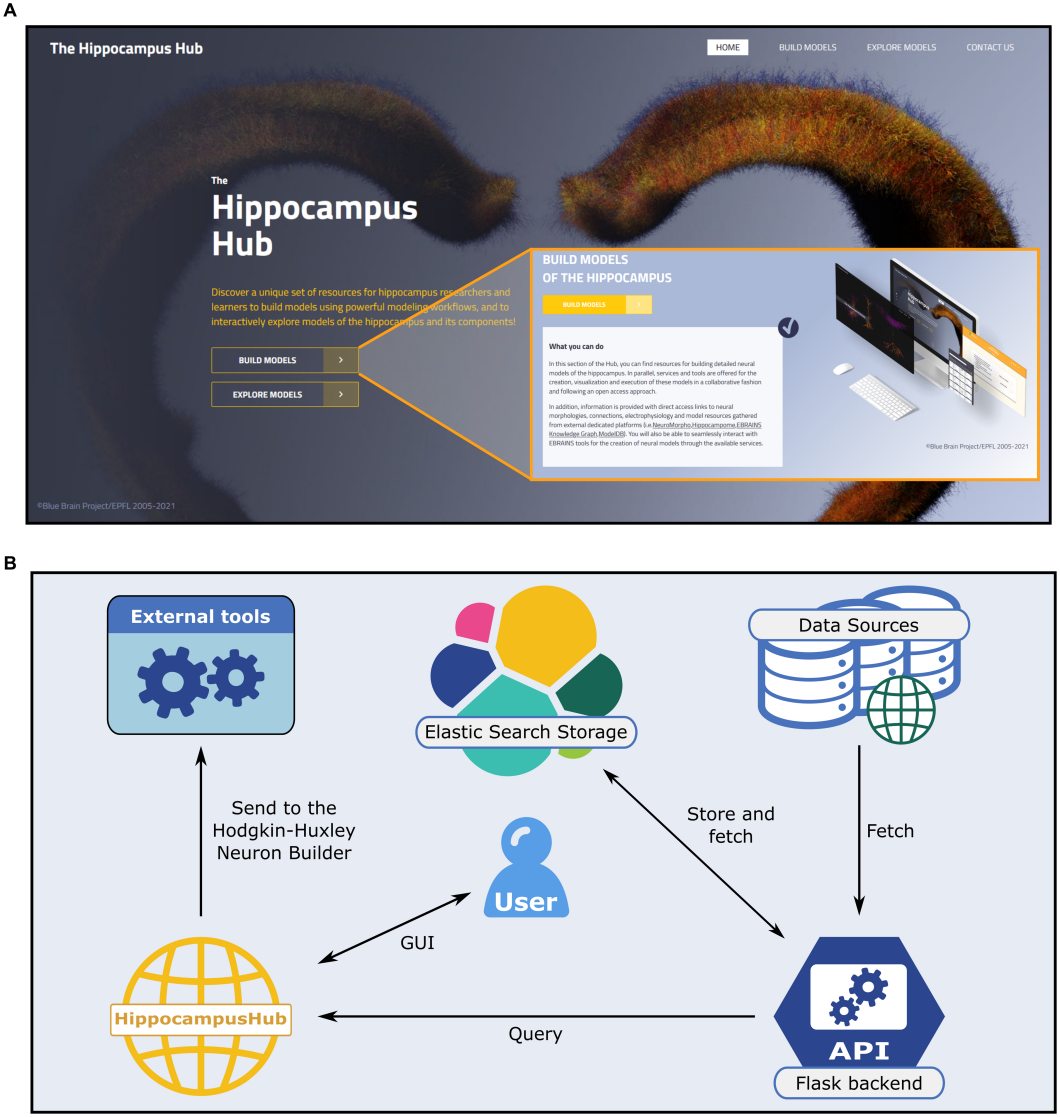
The Hippocampus Hub landing page and software architecture. **(A)** The hub main banner provides direct access to the Build and Explore sections as well as links to the portal subpages via the main menu (top right). A brief description of the purpose of each section (inset) is provided. **(B)** Software architecture of the Build section engine. A Python-based backend (i.e., Flask framework) queries external and internal (i.e., the Explore section content) resources for hippocampus related data and metadata and stores them via the Elastic Search engine on a dedicated virtual machine. The user interactively explores the different sections of the hub and collects the data of interest prior to transferring them to the Hodgkin-Huxley Neuron Builder, for the optimization and simulation of neural models.

The HH consists of frontend (client-side) and backend (server-side) components that communicate via dedicated REST API calls. The source code of the HippocampusHub is hosted on a public GitHub project,^14^ with the code relative to the Build section being stored on a dedicated repository (i.e., https://github.com/hippocampushub/build). The frontend of the portal (https://www.hippocampushub.eu/build/, see Figure 1A) is built via the JavaScript based environment *React*,^15^ by leveraging the *Next.js* framework,^16^ and styled via Cascading Style Sheets (CSS) files. It is deployed on GitHub, through the HTTPS secure protocol, which serves the requests performed to the public *hippocampushub.eu* domain (a redirection from *hippocampushub.eu* to *https://hippocampushub.github.io/* is performed transparently via DNS configuration). The backend of the HH Build section: fetches metadata from sources, provides the information to be displayed to the frontend, is implemented via the Python-based Flask micro-web framework^17^ and relies on the WSGI web server^18^ and on the NGINX proxy manager.^19^ It is packaged and deployed via a Docker container^20^ and hosted on a Virtual Machine (VM), in the CINECA supercomputer,^21^ which features 4 Virtual CPUs, 30GB RAM, and 130GB storage space, accessible and managed via Openstack.^22^ The backend services are available at a registered public domain^23^ and a dedicated Swagger page shows all the available APIs^24^ and how to use them.

In order to guarantee ease of access and high throughput query operations we use, in the backend web server, the Elasticsearch engine^25^ to store all the information (i.e., the metadata) retrieved from the external portals (see Figure 1B) and provided to the HH Build section GUI. Such information are saved in the *.json* format.^26^ The Elasticsearch storage is populated by querying the source repositories via the APIs made available by the creators or through *ad hoc* developed web scraping code. More specifically, the access to the Explore section of the HH is performed through the Blue Brain Nexus platform,^27^ based on an Elasticsearch database. The query is implemented via the *elasticsearch* and *elasticsearch_dsl* Python libraries. The interaction with Neuromorpho is realized through the API available at https://neuromorpho.org/api.jsp. As for the model components available on ModelDB, biophysical mechanisms of complete models are fetched with individual links to open them on the source web pages. Finally, relevant details of the hippocampal circuitry are retrieved from Hippocampome available API endpoint. The filters and keywords used in the queries performed on all the remote portals are reported in the Supplementary materials.

The source repositories are queried automatically and periodically (i.e., every few hours) to track any content update on the linked resource. Fetched data are homogenized, in order to ease their manipulation and transfer to the frontend, following a common format (see Supplementary materials). Thanks to this workflow, the users are constantly provided with the same items available on the original portals.

### 2.3. The EBRAINS Hodgkin-Huxley Neuron Builder

Data and models provided in the HH Build section can be selected and used to construct a data-driven single cell NEURON model, optimize its parameters and explore its behavior via *in silico* simulations, through the HHNB,^28^ which is part of the ecosystem of tools and services available through the EBRAINS Research Infrastructure and has been thoroughly described in a previous work (Bologna et al., 2022). Briefly, the HHNB consists in a full stack web application that manages multi-user workflows that include three steps. First, the users perform the extraction of electrophysiological features from neural recordings, via the NeuroFeatureExtract tool (Bologna et al., 2021), which is based on the eFEL Python library^29^ and allows visual inspection and selection of individual traces prior to the extraction process. Then, the selection of a single neuron model, implemented in NEURON, from the MC^30^ and/or the construction of a model via the upload of individual NEURON *.mod* and parameter files is done. Any *.mod* file can be uploaded by the users, but the content of these files must be mirrored in the *parameters.json* file (part of the BluePyOpt execution file ensemble and editable through the HHNB interface), where the neural mechanisms and the channel distributions are specified (see (Bologna et al., 2022) and (Geit et al., 2016) for further details). Finally, the optimization of the model parameters is performed via the genetic-algorithm-based Python library BluePyOpt (Geit et al., 2016); this process is run on HPC systems upon configuration of both the optimization algorithm parameters (e.g., number of generations) and the requested system resources (e.g., number of HPC nodes); (4) the simulation of the optimized is launched model in the BlueNeuronAsAService (BlueNaaS, https://ebrains-cls-interactive.github.io/online-use-cases.html#/single_cell_insilico_experiments) simulation environment. Concerning the optimization process, which is the core feature of the HHNB and requires supercomputer resources to be carried out, we draw the reader’s attention to the opportunity, for the users, to submit jobs with no need for personal or institutional account on HPC systems. In fact, we have built an *ad hoc* utility, the HHNB Service Account that allows to submit the model optimization jobs and manage individual quotas on behalf of the users, who are only requested to own EBRAINS credentials. In addition, those who possess an account on either the CSCS-DAINT or the NeuroScience Gateway (NSG), can use their own resources on these two systems for the optimization process -see (Bologna et al., 2022) for more details.

It is important to stress the complementarity of the HHNB and the HH resources. In fact, while the HHNB allows to launch and monitor the optimization workflows, and gives the opportunity to modify, or create from scratch, individual neuron models of hippocampal cell, the model components (i.e., morphology, *.mod* and electrophysiology files) available to the users are limited and the use of different files, hosted on different platforms, requires manual search, modification, and upload. Instead, the HH Build section searches *de facto* standard and curated repositories of neural data and model, with no need for any user’s manual intervention, and provides a plethora of modeling components that are automatically filtered to show only those related to the hippocampal brain region. Such resources are then displayed and linked on the HH Build pages in order to be used, in a transparent way, in the HHNB optimization workflow.

### 2.4. Usage

**Figure 2:**
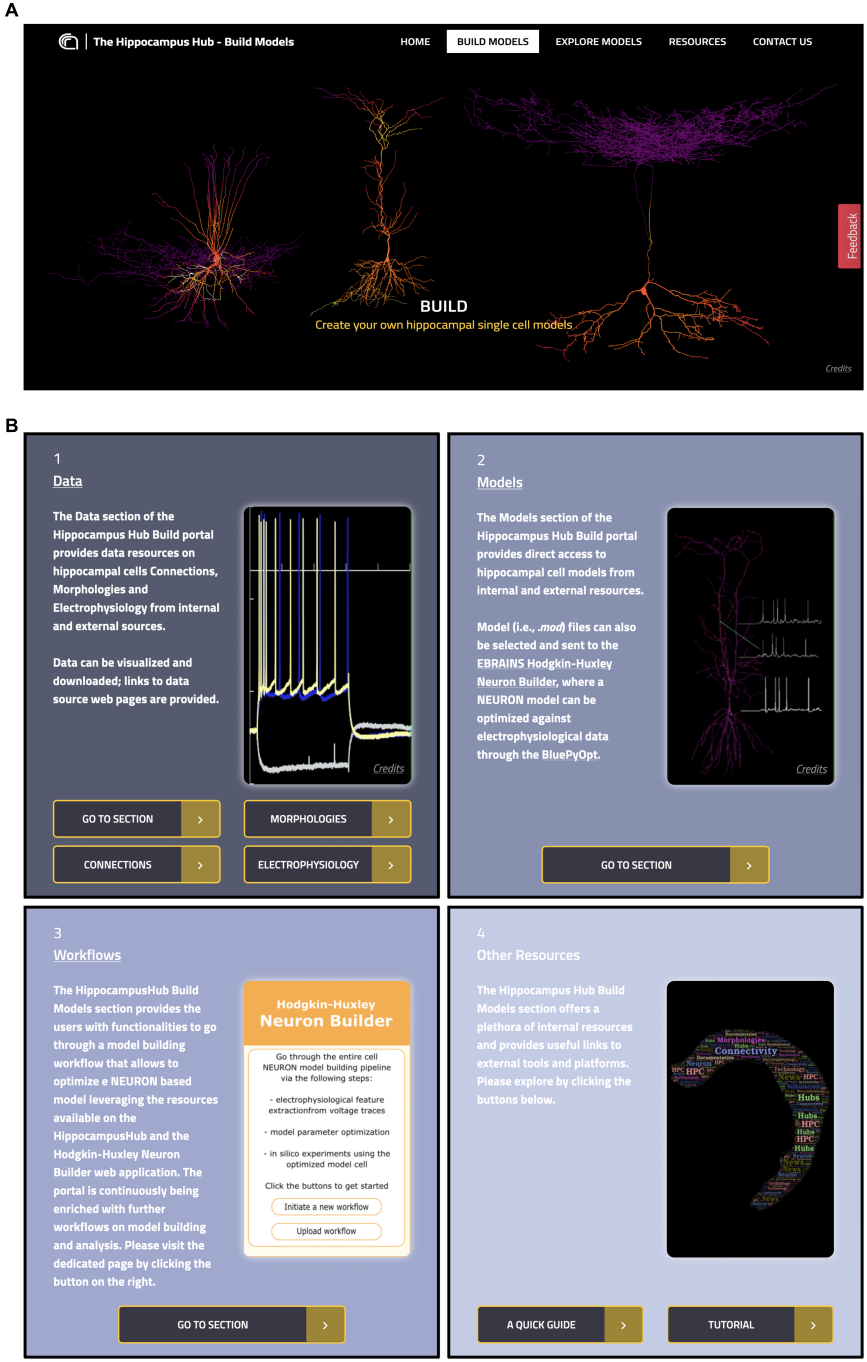
The Hippocampus Hub Build section interface. **(A)** Main banner of the Build section landing page. **(B)** The four panels giving access to the Build section contents: 1 and 2, links to Data and Models pages respectively, with metadata and access urls to the original resources; 3, direct access to external workflows for hippocampus model building and simulation and 4, links to related resources.

The Build section of the HH allows users to access hippocampus-related computational resources available in open online repositories and visualize the relevant metadata, including urls of the original resource website and scientific articles, thumbnails of brain structure images or sketches, neural activity, and 3D representations. Through a user-friendly interactive interface, the dataset can be filtered by specific fields and selected for further use in the model optimization workflow (see section *The EBRAINS Hodgkin-Huxley Neuron Builder*). The section includes four main subparts: the Data and Models pages, which provide access to the actual hippocampal resources, the Workflows page, containing links to external tools and services for hippocampus related model building and simulation and the Other Resources page, which includes a user tutorial, policy, and terms of use (see Figure 2).

#### 2.4.1. Data

**Figure 3:**
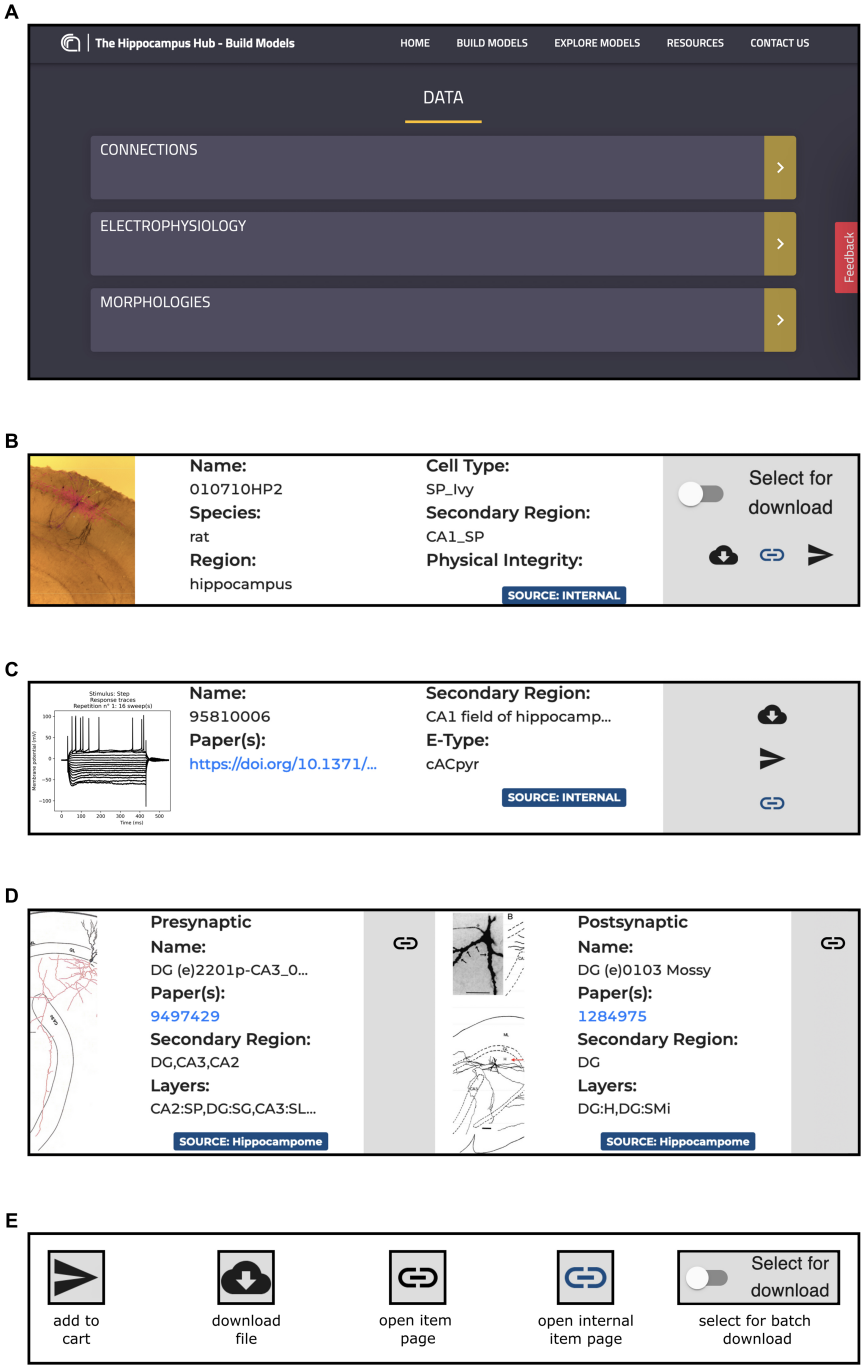
The Build section Data section content. **(A)** Main board with direct links to the Connections, Morphologies and Electrophysiology pages. **(B)** Example of panel containing metadata and links for an item of the Morphologies resource including, information on the Species, Regions and Cell Type of the related morphology, together with direct link to the original resource page and buttons for downloading the morphology file and for adding it to the HHNB cart. **(C)** Example of panel containing metadata and links for an item of the Electrophysiology resources, including metadata on the relative Region and Electrical Type, and links to the original page resource and related publication. All the panels contain magnifiable thumbnails of the related item. **(D)** Example of a panel containing metadata and links for an item of the Connections resources: on the left side, the presynaptic neuron information is contained, including metadata on the concerned brain region and layer, and link to the relevant publication and to the original resource page (i.e., hippocampome.org); **(E)** Button legend for item related actions. Internal pages are those belonging to the Hippocampus Hub Explore section.

**Figure 4:**
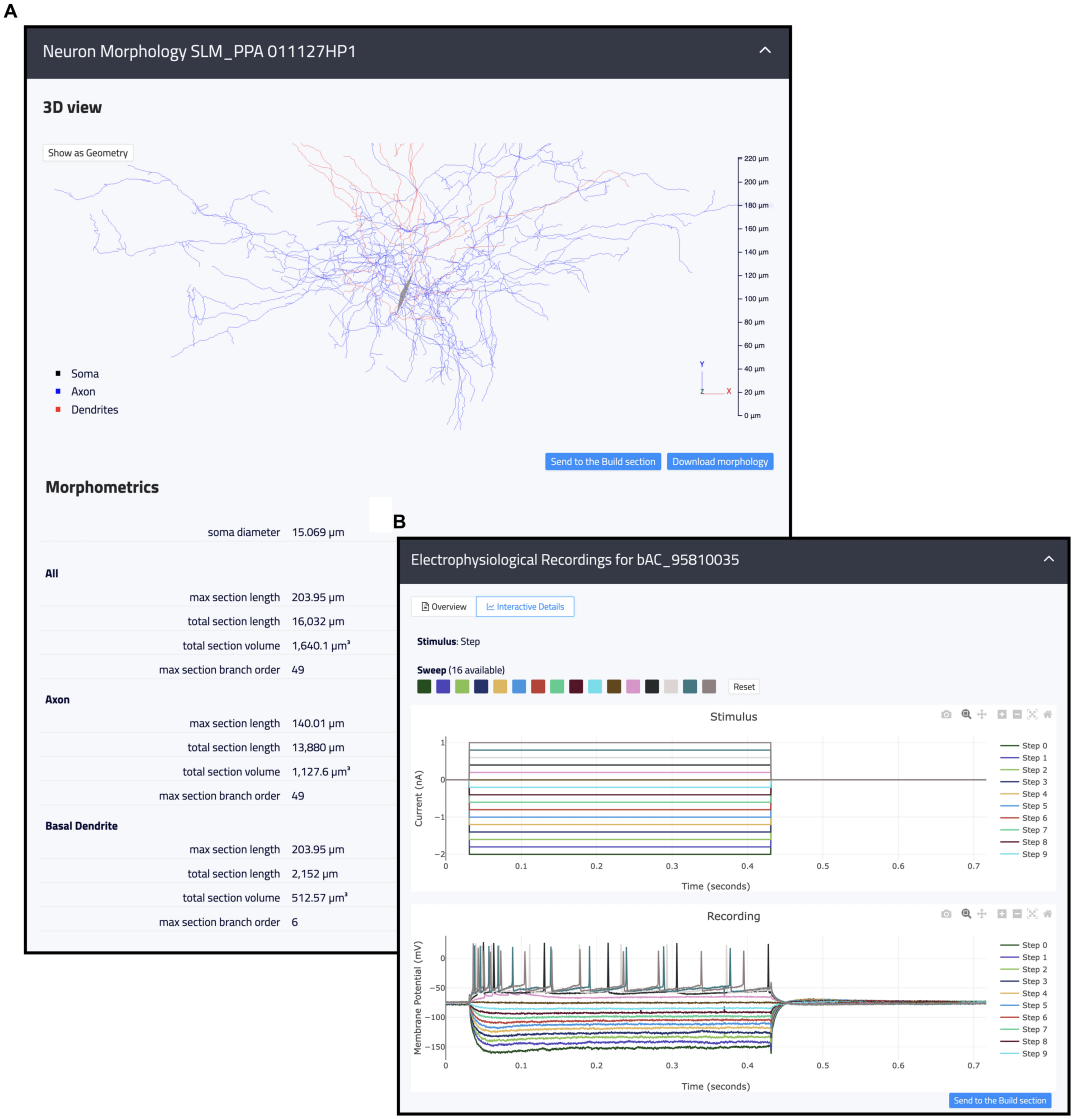
Example of internal resources available in the Hippocampus Hub Explore section. **(A)** 3D interactive view of a hippocampal morphology with related metadata on section lengths, volumes, and branch orders for the entire morphology as well as individual neurite types (i.e., axon and dendrites). **(B)** 2D interactive plots of an individual electrophysiological recording including fourteen traces acquired upon a step stimulation protocol. Trace lines can be zoomed in and out and selectively displayed. Both the morphologies and the traces can be contextually opened in the Build section and selected for the optimization workflow.

This page is made up of three main components: Morphologies, Electrophysiology and Connections (see Figure 3). Each item has a dedicated panel that reports relevant information and links. The Morphologies page currently provides access to 97 items that present images of the digital reconstructions, together with their names, cell types, regions, and physical integrity. Morphologies can be downloaded individually or selected for batch download, opened in the original source page, visualized via a 3D viewer and zoomed in and out (morphometrics details are also provided such as section lengths and volumes), and added to the HHNB cart for use in the model building workflow (only one morphology can be selected for this purpose, see Figure 4A). In the Electrophysiology page, 176 single cell recordings are currently available. These are fetched from either Hippocampome or the HH Explore section and can be selected for individual or batch download. As for the morphologies, individual or multiple items can be added to the HHNB cart (this action is only allowed for files residing in the Explore section of the HH, currently 78 items). Electrophysiological traces are also displayed in interactive frames where individual activity and stimulus traces can be selected (see Figure 4B). Currently, all the internal experimental recordings are contributed by Alex Thomson’s group at UCL and belong to four different electrical types (i.e., e-types), classified upon the Petilla convention (Ascoli et al., 2008): (1) cAC (continuous accommodating cells), (2) bAC (bursting accommodating cells), (3) cNAC (continuous non-accommodating cells), and (4) cACpyr (continuous accommodating pyramidal cells). Finally, the Connections page currently provides access to 195 items, fetched from the Hippocampome repository. For each connection, metadata on the presynaptic and postsynaptic neurons are given that indicate the neuron name, layer and region, the url of the original item in the Hippocampome website and the scientific article in which the connection type was investigated. Connection items are displayed for providing an overall picture (and individual connection details) of the state of the art of hippocampal region connectivity studies; they do not refer to any specific file that can be used for the single neuron model building, though; hence, no button is available to add Connections items to the HHNB cart.

#### 2.4.2. Models

**Figure 5:**
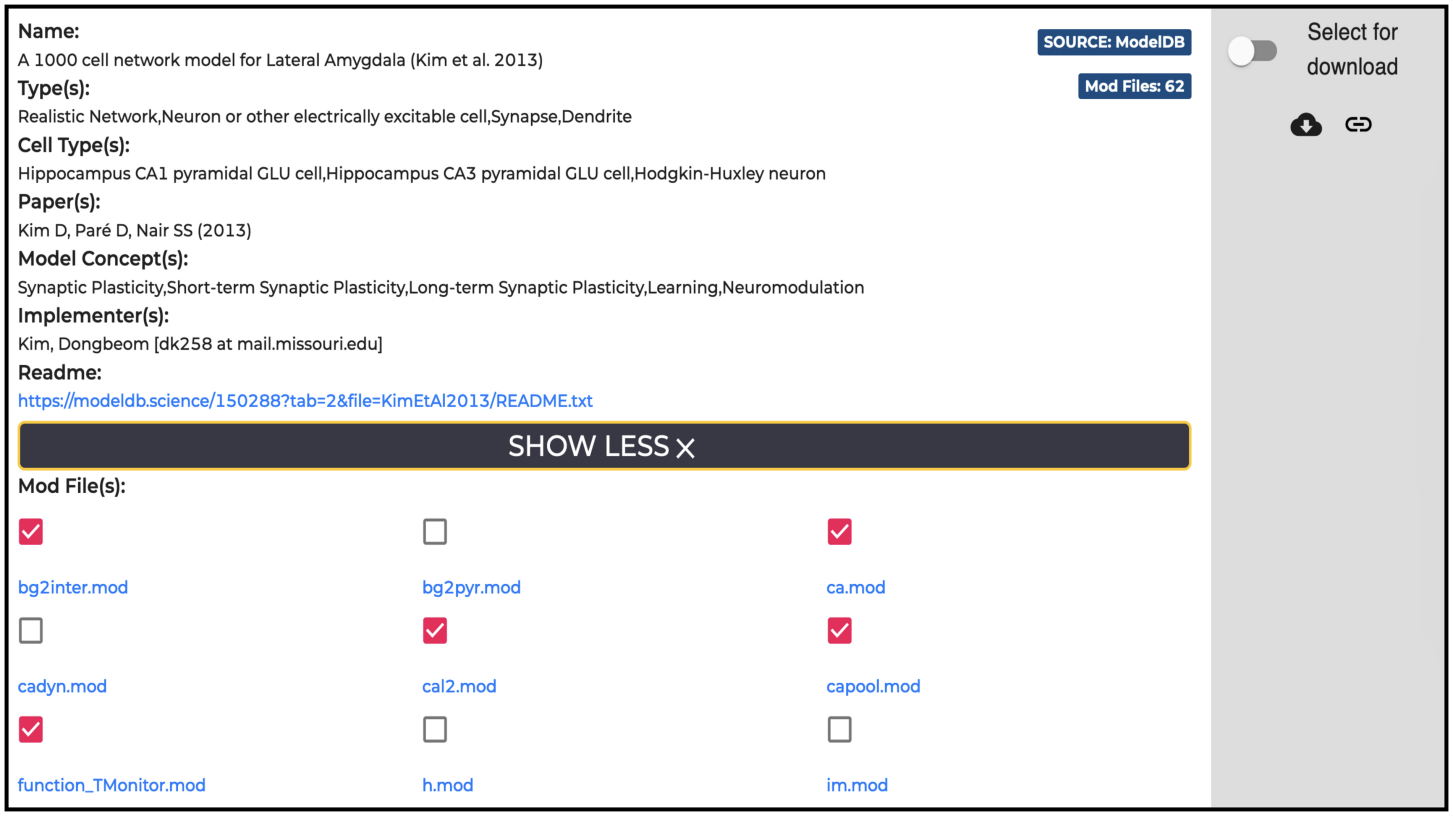
Example of a Model item panel. In addition to the metadata and link related to the model, the list of all the NEURON *.mod* files the model is built with is displayed. Individual *.mod* files (from a single or multiple models) can be added to the Hodgkin-Huxley Neuron Builder cart for model optimization and simulation.

All the entries (135 at the time of writing) available in the Models section are hosted in the ModelDB portal. For each model, the HH page displays name, type, cell type, relevant publications, model concept and implementers, together with a “Download” and a “Add to cart” buttons for downloading and/or adding individual *.mod* files to the workflow, respectively. These are text files containing instructions, written in the NEURON MODeling Language (NMODL, https://bluebrain.github.io/nmodl/html/language.html), that specify the neural mechanisms underlying the implemented model (e.g., biophysical mechanisms such as ligand/voltage-gated ion channels or ion accumulation mechanisms, synaptic plasticity mechanisms or current/voltage sources). All the *.mod* files describing each model are listed (see Figure 5). These files are not editable/visible on the HH page, but we provide a link to individual items on ModelDB (taking advantage of the services offered by the hosting platform), where they are accessible for download and visualization and where a downloadable package of the full model is provided. In this way, the users can explore the content of each file (such as ion species, kinetics, and parameters available for optimization) prior to its use in the optimization workflow. In case the need for new or modified files arises, these can be uploaded via the HHNB user interface. In order to ease the visualization of the *.mod* files, we are considering the use of a dedicated nmodl parser made available by the BlueBrain.^31^

#### 2.4.3. Workflows

**Figure 6:**
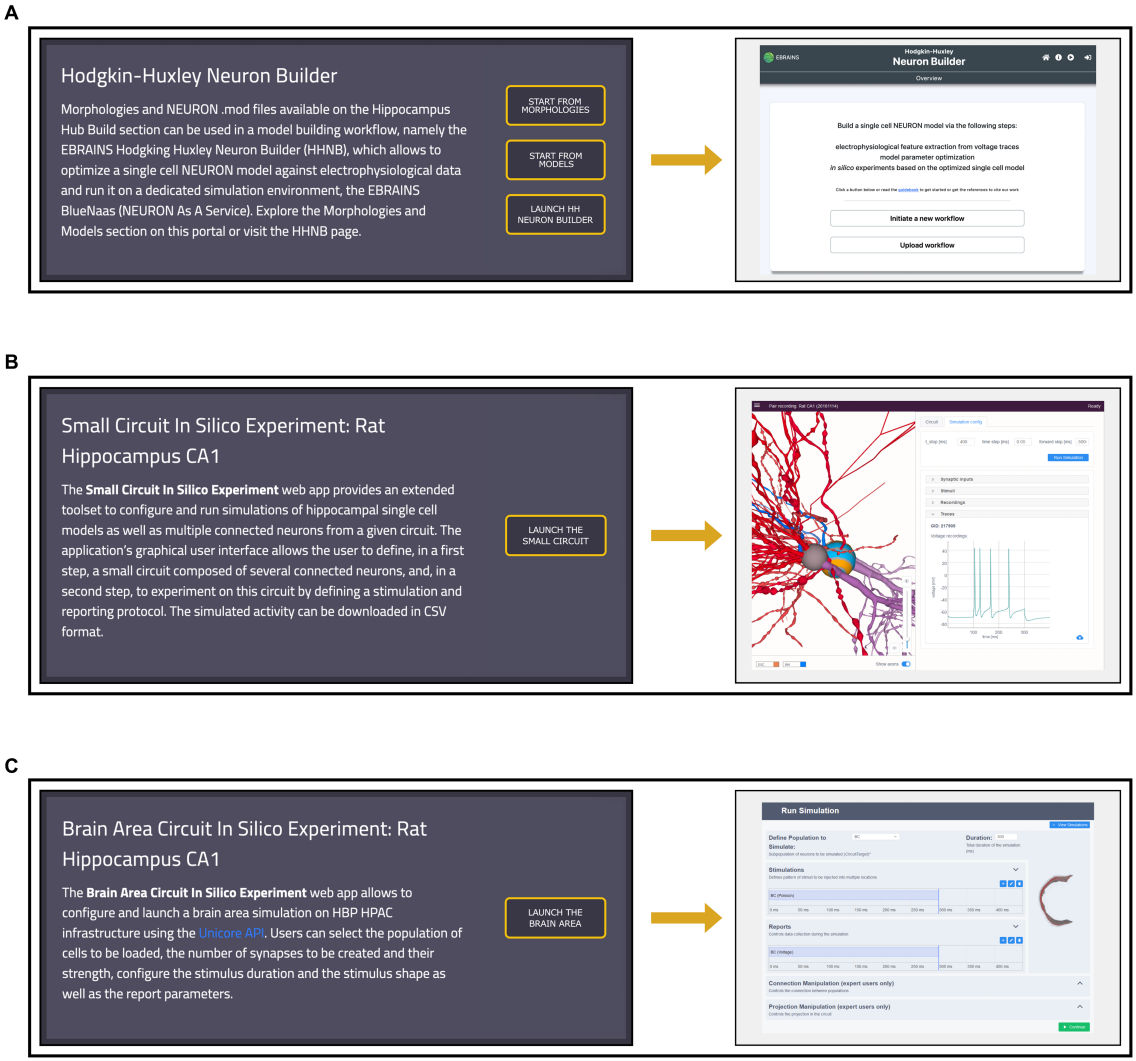
Workflows page overview. **(A)** Left: access to the Morphologies and Models pages, from which data for the single neuron optimization can be added to the cart, and direct link to the Hodgkin-Huxley Neuron Builder (HHNB). Right: HHNB homepage. **(B)** Left: short description and access button for the Small Circuit *in Silico* Experiment simulation tool. Right: Small Circuit simulation web application homepage; **(C)** Left: short description and access button for the Brain Area Circuit *in Silico* Experiment simulation tool. Right: Brain Area simulation web application homepage.

The HH Build section also features a page providing direct links to model building and simulation frameworks (see Figure 6). The HHNB (see section *The EBRAINS Hodgkin-Huxley Neuron Builder*) can be directly accessed, or launched after the items of interest (e.g., morphology, electrophysiology or model files) have been collected in the HHNB cart. Currently, the model building and optimization resources available through the HH are not integrated in any circuit building workflow but exclusively focus on the construction of biophysically detailed NEURON models of single cells. Nonetheless, we believe that researchers investigating data-driven computational models of the hippocampus, might be interested in the behavior of larger scale hippocampal models. For this reason, in the Workflows page, we added two additional links to a small circuit and brain region simulation tools, respectively. The “Small Circuit *in Silico* Experiment: Rat Hippocampus CA1” allows the construction and simulation of a small circuit of rat CA1 cells. Users build the small circuit by selecting individual cells from a reconstructed atlas (Akram et al., 2018), via an interactive graphical interface, which provides morphological, anatomical and electrical information on individual cells. Once the small circuit is built, the simulation and stimulus parameters are set and the *in silico* experiment launched. The resulting simulated activity can be downloaded in text format for further processing and analysis (not shown). The “Brain Area Circuit *in Silico* Experiment: Rat Hippocampus CA1” allows the users to run a simulation of an entire hippocampal cell population, which can be interactively selected based on cell properties (e.g., cell location, electrical type). After designing the stimulus pattern to be delivered (multiple site stimulations are allowed), the simulation is run on the CSCS HPC system, via a service-account-based job submission (see section *The EBRAINS Hodgkin-Huxley Neuron Builder*). Once the simulation is completed, results are fetched and analyzed following the user’s settings (not shown). For details on the hippocampus model implemented, see (Ecker et al., 2020) and the relative Live Paper.^32^

### 2.5. An example of model building and optimization workflow

**Figure 7:**
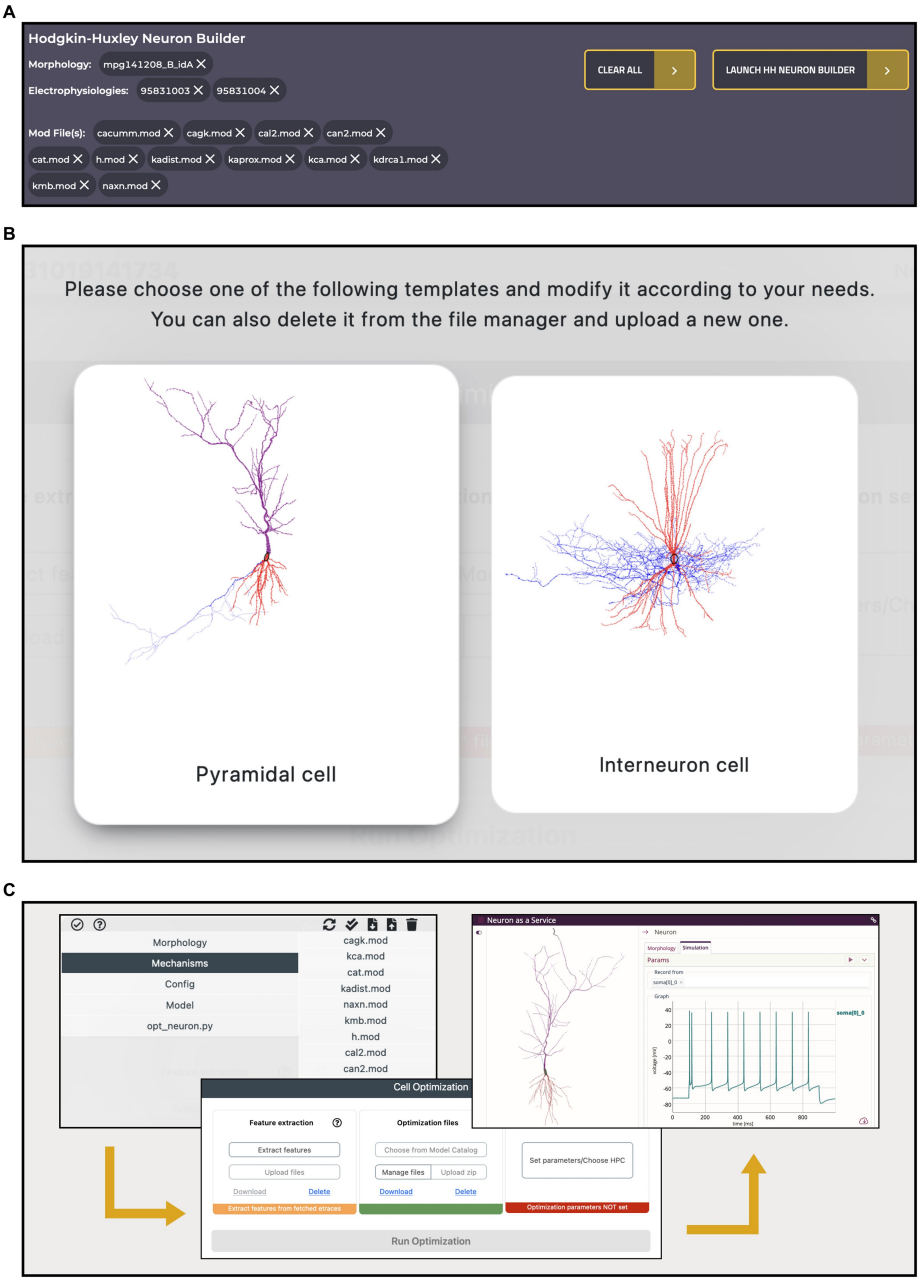
Hodgkin-Huxley Neuron Builder cart and workflow. **(A)** Cart banner containing all the items selected by the user and to be transferred to the Hodgkin-Huxley Neuron Builder for optimizing a single NEURON cell, based on the chosen morphology, and against the selected electrophysiology traces features. A single morphology file can be chosen for the optimization process while multiple selections are allowed for both neural recordings and NEURON *.mod* files. **(B)** Selection panel for the type of neuron model the user wants to optimize. Depending on the selection, a specific *parameters.json* template file, required by the BluePyOpt optimization library, is loaded, and can be modified by the users. **(C)** Optimization workflow performed through the Hodgkin-Huxley Neuron Builder: after users have verified and/or modified the model according to their needs (left), extracted the features of interest and submitted the optimization job to the HPC system (center), they can fetch and simulate the optimized model via the BlueNaas framework (right).

A key feature of the HH Build section is the possibility to collect resources from several, comprehensive, remote and local inventories and use them in a model building and optimization workflow, thanks to its integration with the HHNB. This is realized via a dedicated cart that collects all the items selected by the users as they explore the HH portal (see Figure 7A). Such a cart is implemented via a dedicated panel from which individual items can be added or removed at any time.

To illustrate an example of workflow, we went through the data and model selection in the Electrophysiologies, Morphologies and Models pages and seamlessly transferred all the relevant information (i.e., the urls of the original source files) to the HHNB, for the feature extraction, model optimization and simulation steps. The integration with the HHNB is done transparently, thanks to *ad hoc* developed APIs that allow seamless data/models moving and use. In order to demonstrate the ease-of-use of the portal, highlight the integration of the available resources and guide the user through the component-by-component model construction, we assembled the same model that we adopted in our previous work. In the latter, the user intervention was limited to the selection of the traces and features of interest in the feature extraction phase, the choice of a fully-fledged model to be optimized and the setting of the optimization parameters. Here, we go through the model construction by showing that the user can not only filter, visualize, interact with, and select the morphology and recordings of interest but also individually collect several *.mod* files (from different items, if needed), add all the resources to the cart and carry out a complete model optimization workflow (see Figures 7B,C and the dedicated Tutorial in the HH Build section page). In Table 1, we report the details of the data and model components selected in the workflow.

**Table 1:** Data selected in the HH portal for the construction of the model and parameters chosen in the HHNB for the feature extraction and model optimization.

**Hippocampus Hub**
**Morphology** *Item name*: mpg141208_B_idA *Url*: https://www.hippocampushub.eu/build/data/morphology
**Electrophysiologies** *Item names*: 95831003, 95831004 *Url*: https://www.hippocampushub.eu/build/data/electrophysiology
**Model** *Item name*: Circadian rhythmicity shapes astrocyte morphology and neuronal function in CA1 (McCauley et al. 2020) *.mod files*: cacumm.mod, cagk.mod, cal2.mod, can2.mod, cat.mod, h.mod, kadist.mod, kaprox.mod, kca.mod, kdrca1.mod, kmb.mod, naxn.mod *Url*: https://www.hippocampushub.eu/build/models
**Hodgkin-Huxley Neuron Builder**
**Stimulus amplitudes** 1 nA, 0.8 nA, 0.6 nA, −0.2 nA, −0.6 nA, −0.8 nA.
**Extracted features** “inv_fifth_ISI”, “inv_first_ISI”, “inv_fourth_ISI”, “inv_last_ISI”, “inv_second_ISI”, “inv_third_ISI”, “mean_frequency”, “Spikecount”, “steady_state_voltage”, “voltage_base”.
**Optimization settings** # Gen: 24; Offspring: 10; # Nodes 6; # Cores: 24; Runtime: 2; HPC system: CSCS-DAINT.

## 3. Discussion

In the framework of the EBRAINS Research Infrastructure, the HBP (Amunts et al., 2016) and the EBRAINS-Italy (ebrains-italy.eu) projects, we have developed an online resource, the Build section of the Hippocampus Hub, with the goal of creating a reference portal for the wider scientific community interested in building data-driven, biophysically and morphologically detailed models of single hippocampal cells, by collecting computational resources available online on several specialized platforms (e.g., NeuroMorpho, KG, ModelDB, Hippocampome,) (Wheeler et al., 2015; McDougal et al., 2017; Akram et al., 2018). The entries accessible on the portal include electrophysiology, morphology, connectivity data, and models, and are constantly and transparently updated, in accordance with the original resources. Every entry displays relevant information (e.g., brain region, animal species, reference scientific papers, original source url) and, depending on the data type, allows visual inspection via 2D images or 3D representations. A unique feature of the HH Build section is the integration with the HHNB (Bologna et al., 2022) that offers a friendly interface and the needed computational resources for the realization of a complete data-driven model construction, optimization and simulation workflow, for models implemented in the NEURON simulation environment (Hines and Carnevale, 1997). HH visitors can freely explore the portal and select the data they want to use in the model building process, by collecting them in the HHNB cart, similarly to filling a cart in an e-shop: thanks to *ad hoc* developed REST APIs, the urls of the selected resources are transparently sent to the HHNB where a new workflow is initialized with the chosen items. Here, the HHNB interface allows to explore, modify, or remove the selected files, while giving the opportunity to add new ones, for the feature extraction and model construction.

While the presented resource already allows to go through a complete single cell model creation workflow, several improvements and upgrades are currently being planned to provide the users with a richer and more comprehensive research framework. Being the software architecture and the used technologies brain region agnostic, with a relatively little effort the developed platform can be mirrored onto other hubs that focus on different brain regions (e.g., cerebellum). Also, as the KG and the referenced inventories increase their offer, in terms of data and models, we plan to extend the HH Build section accordingly, by including new type of data (e.g., novel morphologies or recording type, such as the NWB format) and models (e.g., modules from neural models other than the NEURON ones) in the model construction process. We foresee to tighten the integration with the MC, as well, by listing it in the set of available online repositories from which to fetch models of interest (including those previously optimized) and allowing the users to store models directly from the HH pages. At the moment, the entire workflow aims at creating a model *.zip* file, ready to be fed to the BluePyOpt optimizer, based on the NEURON modeling language. A possible extension of this implementation would be to include the creation/modification of models expressed in the NeuroML or SONATA languages (this would require a modification of the HHNB web application too, with respect to the model formatting and submission to HPC via the service account utility).

Finally, we plan to build a provenance engine to keep track of the choices and operations performed by the users. A first step would be storing the items selected from the HH GUI (e.g., morphologies and *.mod* files) in the users’ GitHub (or other platforms) repositories. Such a feature would create a bridge with other resources such as the OSB (or, in the future, NetPyNE or the EBRAINS simulation tools), where users’ data/code repositories are monitored and integrated to be used through development, analysis and visualization applications (e.g., NWB Explorer, JupyterLab). Such an improvement would help to foster open and community science in the field of neural modeling.

## Data availability statement

The datasets presented in this study can be found in online repositories. The names of the repository/repositories and accession number(s) can be found at: https://www.hippocampushub.eu/.

## Author contributions

LB: Conceptualization, Software, Writing – original draft, Writing – review & editing. AT: Software, Writing – review & editing. RS: Software, Writing – review & editing. AR: Conceptualization, Writing – review & editing. FS: Conceptualization, Writing – review & editing. MM: Conceptualization, Funding acquisition, Supervision, Writing – review & editing.

## Footnotes

^1^ https://neuroml-db.org/

^2^ https://alleninstitute.org/

^3^ https://openneuro.org/

^4^ https://neuromorpho.org/

^5^ https://figshare.com

^6^ https://datadryad.org/

^7^ https://www.ebrains.eu/

^8^ https://live-papers.brainsimulation.eu/

^9^ https://wiki.ebrains.eu/

^10^ https://www.ebrains.eu/modelling-simulation-and-computing/simulation

^11^ https://www.hippocampushub.eu/build/

^12^ https://neuromorpho.org/

^13^ https://hippocampome.org/

^14^ https://github.com/hippocampushub

^15^ https://react.dev/

^16^ https://nextjs.org/

^17^ https://palletsprojects.com/p/flask/

^18^ https://wsgi.readthedocs.io

^19^ https://nginxproxymanager.com/

^20^ https://www.docker.com/

^21^ https://www.cineca.it/en

^22^ https://www.openstack.org/

^23^ https://hh-build.ebrains-italy.eu/

^24^ https://hh-build.ebrains-italy.eu/apidocs/

^25^ https://www.elastic.co/

^26^ https://www.json.org

^27^ https://bbp.epfl.ch/nexus/web/

^28^ https://hhnb.ebrains-italy.eu/hh-neuron-builder/

^29^ https://pypi.org/project/efel/

^30^ https://model-catalog.brainsimulation.eu/

^31^ https://github.com/BlueBrain/nmodl

^32^ https://live-papers.brainsimulation.eu/#2020-ecker-et-al
