# Supplementary material for: Online interoperable resources for building hippocampal neuron models via the Hippocampus Hub
Source: Front Neuroinform. 2023 Nov 1;17:1271059. doi: 10.3389/fninf.2023.1271059 (PMC10646550; doi:10.3389/fninf.2023.1271059)
Supplement: Supplementary file 1 [file Data_Sheet_1.docx]

**Online Interoperable Resources For Building Hippocampal Neuron Models Via The Hippocampus Hub**

Luca Leonardo Bologna^1^, Antonino Tocco^1^, Roberto Smiriglia^1^, Armando Romani^2^, Felix Schürmann^2^, Michele Migliore^1^

^1^Institute of Biophysics, National Research Council, Palermo, Italy,

^2^Blue Brain Project, École Polytechnique Fédérale de Lausanne, Geneva, Switzerland

* Correspondence:
Luca Leonardo Bologna
[lucaleonardo.bologna@cnr.it](mailto:lucaleonardo.bologna@cnr.it)

**Supplementary materials**

In the text below, we provide the API and the filters used for querying the platforms and extract the metadata of interest.

**Neuromorpho**

API used for querying the platform and extract the metadata of interest:

<https://neuromorpho.org/apiReference.html#neuron-filter-query>

Arguments used in the queries:

brain_region: [“hippocampus”],

domain: ["dendrites", "soma", "axon"],

attributes: [“diameter”, “3d”, “angles”]

Physical_Integrity: [“dendrites complete”, “no axon”]

Morphologies available in file formats different from .asc and .swc were disregarded.

**ModelDB**

API used for querying the platform and extract the metadata of interest:

<http://modeldb.science/api/v1/models?modeling_application=NEURON>.

For individual models these endpoints are used:

[http://modeldb.science/api/v1/models/{id}](http://modeldb.science/api/v1/models/%7bid%7d)

[https://senselab.med.yale.edu/modeldb/ShowModel?model={id}](https://senselab.med.yale.edu/modeldb/ShowModel?model=%7bid%7d) (the results from this page are parsed through a web scraping algorithm).

Once the queries are answered, only models of cell type “hippocampus” are kept.

**HH Explore section**

API used for querying the platform and extract the metadata of interest:

<https://bbp.epfl.ch/nexus/v1/views/public/hippocampus-hub>

Arguments used in the queries:

“NeuroMorphology” or “Trace”, for the reconstructed morphology and the neural recordings respectively.

**Hippocampome**

API used for querying the platform and extract the metadata of interest:

<http://hippocampome.org/php/search_engine_json.php?query_str=>

Arguments used in the queries, grouped by item type:

Morphologies:

http://hippocampome.org/php/search_engine_json.php?query_str=Neuron:(Presynaptic:(Markers:(D±:CB OR I±:CB OR D±:CR

 OR I±:CR OR D±:PV OR I±:PV OR D±:Mus2R OR I±:Mus2R OR D±:5HT-3 OR I±:5HT-3 OR D±:Gaba-a-alpha OR I±:Gaba-a-alpha OR D±:mGluR1a OR I±:mGluR1a OR D±:vGluT3 OR I±:vGluT3 OR D±:CCK OR

 I±:CCK OR D±:SOM OR I±:SOM OR D±:nNOS OR I±:nNOS OR D±:PPTA OR I±:PPTA OR D±:vGluT2 OR I±:vGluT2 OR D±:CGRP OR I±:CGRP OR D±:mGluR2/3 OR I±:mGluR2/3 OR D±:mGluR5 OR I±:mGluR5 OR D

±:Prox1 OR I±:Prox1 OR D±:GABAa \delta OR I±:GABAa \delta OR D±:MUS1R OR I±:MUS1R OR D±:Mus3R OR I±:Mus3R OR D±:Mus4R OR I±:Mus4R OR D±:Cam OR I±:Cam OR D±:AMPAR 2/3 OR I±:AMPAR 2/

3 OR D±:Disc1 OR I±:Disc1 OR D±:BONG OR I±:BONG OR D±:p-CREB OR I±:p-CREB OR D±:Neuropilin2 OR I±:Neuropilin2 OR D±:mGluR1 OR I±:mGluR1 OR D±:Caln OR I±:Caln OR D±:vGlut1 OR I±:vGl

ut1 OR D±:mGluR2 OR I±:mGluR2 OR D±:mGluR3 OR I±:mGluR3 OR D±:GABAa\alpha 2 OR I±:GABAa\alpha 2 OR D±:GABAa\alpha 3 OR I±:GABAa\alpha 3 OR D±:GABAa\alpha 4 OR I±:GABAa\alpha 4 OR D

±:GABAa\alpha 5 OR I±:GABAa\alpha 5 OR D±:GABAa\alpha 6 OR I±:GABAa\alpha 6 OR D±:GABAa\beta 1 OR I±:GABAa\beta 1 OR D±:GABAa\beta 2 OR I±:GABAa\beta 2 OR D±:GABAa\beta 3 OR I±:GAB

Aa\beta 3 OR D±:GABAa\gamma 1 OR I±:GABAa\gamma 1 OR D±:GABAa\gamma 2 OR I±:GABAa\gamma 2 OR D±:mGluR5a OR I±:mGluR5a OR D±:GlyT2 OR I±:GlyT2 OR D±:mGluR7a OR I±:mGluR7a OR D±:mGlu

R8a OR I±:mGluR8a OR D±:vAChT OR I±:vAChT OR D±:AChE OR I±:AChE OR D±:Kv3.1 OR I±:Kv3.1 OR D±:Cx36 OR I±:Cx36 OR D±:AR-beta1 OR I±:AR-beta1 OR D±:AR-beta2 OR I±:AR-beta2 OR D±:TH O

R I±:TH OR D±:mGluR4 OR I±:mGluR4 OR D±:CXCR4 OR I±:CXCR4 OR D±:GABA-B1 OR I±:GABA-B1 OR D±:GluA2 OR I±:GluA2 OR D±:GluA1 OR I±:GluA1 OR D±:GluA3 OR I±:GluA3 OR D±:GluA4 OR I±:GluA

4) OR Morphology:(Axons:DG:???? OR Axons:CA3:????? OR Axons:CA2:???? OR Axons:CA1:???? OR Axons:SUB:??? OR Axons:EC:?????? OR Soma:DG:???? OR Soma:CA3:????? OR Soma:CA2:???? OR Som

a:CA1:???? OR Soma:SUB:??? OR Soma:EC:?????? OR Dendrites:DG:???? OR Dendrites:CA3:????? OR Dendrites:CA2:???? OR Dendrites:CA1:???? OR Dendrites:SUB:??? OR Dendrites:EC:??????)) A

ND Postsynaptic:(Markers:(D±:CB OR I±:CB OR D±:CR OR I±:CR OR D±:PV OR I±:PV OR D±:Mus2R OR I±:Mus2R OR D±:5HT-3 OR I±:5HT-3 OR D±:Gaba-a-alpha OR I±:Gaba-a-alpha OR D±:mGluR1a OR

I±:mGluR1a OR D±:vGluT3 OR I±:vGluT3 OR D±:CCK OR I±:CCK OR D±:SOM OR I±:SOM OR D±:nNOS OR I±:nNOS OR D±:PPTA OR I±:PPTA OR D±:vGluT2 OR I±:vGluT2 OR D±:CGRP OR I±:CGRP OR D±:mGluR

2/3 OR I±:mGluR2/3 OR D±:mGluR5 OR I±:mGluR5 OR D±:Prox1 OR I±:Prox1 OR D±:GABAa \delta OR I±:GABAa \delta OR D±:MUS1R OR I±:MUS1R OR D±:Mus3R OR I±:Mus3R OR D±:Mus4R OR I±:Mus4R O

R D±:Cam OR I±:Cam OR D±:AMPAR 2/3 OR I±:AMPAR 2/3 OR D±:Disc1 OR I±:Disc1 OR D±:BONG OR I±:BONG OR D±:p-CREB OR I±:p-CREB OR D±:Neuropilin2 OR I±:Neuropilin2 OR D±:mGluR1 OR I±:mG

luR1 OR D±:Caln OR I±:Caln OR D±:vGlut1 OR I±:vGlut1 OR D±:mGluR2 OR I±:mGluR2 OR D±:mGluR3 OR I±:mGluR3 OR D±:GABAa\alpha 2 OR I±:GABAa\alpha 2 OR D±:GABAa\alpha 3 OR I±:GABAa\alp

ha 3 OR D±:GABAa\alpha 4 OR I±:GABAa\alpha 4 OR D±:GABAa\alpha 5 OR I±:GABAa\alpha 5 OR D±:GABAa\alpha 6 OR I±:GABAa\alpha 6 OR D±:GABAa\beta 1 OR I±:GABAa\beta 1 OR D±:GABAa\beta

2 OR I±:GABAa\beta 2 OR D±:GABAa\beta 3 OR I±:GABAa\beta 3 OR D±:GABAa\gamma 1 OR I±:GABAa\gamma 1 OR D±:GABAa\gamma 2 OR I±:GABAa\gamma 2 OR D±:mGluR5a OR I±:mGluR5a OR D±:GlyT2 O

R I±:GlyT2 OR D±:mGluR7a OR I±:mGluR7a OR D±:mGluR8a OR I±:mGluR8a OR D±:vAChT OR I±:vAChT OR D±:AChE OR I±:AChE OR D±:Kv3.1 OR I±:Kv3.1 OR D±:Cx36 OR I±:Cx36 OR D±:AR-beta1 OR I±:

AR-beta1 OR D±:AR-beta2 OR I±:AR-beta2 OR D±:TH OR I±:TH OR D±:mGluR4 OR I±:mGluR4 OR D±:CXCR4 OR I±:CXCR4 OR D±:GABA-B1 OR I±:GABA-B1 OR D±:GluA2 OR I±:GluA2 OR D±:GluA1 OR I±:Glu

A1 OR D±:GluA3 OR I±:GluA3 OR D±:GluA4 OR I±:GluA4) OR Morphology:(Axons:DG:???? OR Axons:CA3:????? OR Axons:CA2:???? OR Axons:CA1:???? OR Axons:SUB:??? OR Axons:EC:?????? OR Soma:

DG:???? OR Soma:CA3:????? OR Soma:CA2:???? OR Soma:CA1:???? OR Soma:SUB:??? OR Soma:EC:?????? OR Dendrites:DG:???? OR Dendrites:CA3:????? OR Dendrites:CA2:???? OR Dendrites:CA1:???? OR Dendrites:SUB:??? OR Dendrites:EC:??????)))

Connections:

http://hippocampome.org/php/search_engine_json.php?query_str=Connection:(Presynaptic:(Markers:(D±:CB OR I±:CB

OR D±:CR OR I±:CR OR D±:PV OR I±:PV OR D±:Mus2R OR I±:Mus2R OR D±:5HT-3 OR I±:5HT-3 OR D±:Gaba-a-alpha OR I±:Gaba-a-alpha OR D±:mGluR1a OR I±:mGluR1a OR D±:vGluT3 OR I±:vGluT3 OR D

±:CCK OR I±:CCK OR D±:SOM OR I±:SOM OR D±:nNOS OR I±:nNOS OR D±:PPTA OR I±:PPTA OR D±:vGluT2 OR I±:vGluT2 OR D±:CGRP OR I±:CGRP OR D±:mGluR2/3 OR I±:mGluR2/3 OR D±:mGluR5 OR I±:mGl

uR5 OR D±:Prox1 OR I±:Prox1 OR D±:GABAa \delta OR I±:GABAa \delta OR D±:MUS1R OR I±:MUS1R OR D±:Mus3R OR I±:Mus3R OR D±:Mus4R OR I±:Mus4R OR D±:Cam OR I±:Cam OR D±:AMPAR 2/3 OR I±:

AMPAR 2/3 OR D±:Disc1 OR I±:Disc1 OR D±:BONG OR I±:BONG OR D±:p-CREB OR I±:p-CREB OR D±:Neuropilin2 OR I±:Neuropilin2 OR D±:mGluR1 OR I±:mGluR1 OR D±:Caln OR I±:Caln OR D±:vGlut1 O

R I±:vGlut1 OR D±:mGluR2 OR I±:mGluR2 OR D±:mGluR3 OR I±:mGluR3 OR D±:GABAa\alpha 2 OR I±:GABAa\alpha 2 OR D±:GABAa\alpha 3 OR I±:GABAa\alpha 3 OR D±:GABAa\alpha 4 OR I±:GABAa\alph

a 4 OR D±:GABAa\alpha 5 OR I±:GABAa\alpha 5 OR D±:GABAa\alpha 6 OR I±:GABAa\alpha 6 OR D±:GABAa\beta 1 OR I±:GABAa\beta 1 OR D±:GABAa\beta 2 OR I±:GABAa\beta 2 OR D±:GABAa\beta 3 O

R I±:GABAa\beta 3 OR D±:GABAa\gamma 1 OR I±:GABAa\gamma 1 OR D±:GABAa\gamma 2 OR I±:GABAa\gamma 2 OR D±:mGluR5a OR I±:mGluR5a OR D±:GlyT2 OR I±:GlyT2 OR D±:mGluR7a OR I±:mGluR7a OR

 D±:mGluR8a OR I±:mGluR8a OR D±:vAChT OR I±:vAChT OR D±:AChE OR I±:AChE OR D±:Kv3.1 OR I±:Kv3.1 OR D±:Cx36 OR I±:Cx36 OR D±:AR-beta1 OR I±:AR-beta1 OR D±:AR-beta2 OR I±:AR-beta2 OR

 D±:TH OR I±:TH OR D±:mGluR4 OR I±:mGluR4 OR D±:CXCR4 OR I±:CXCR4 OR D±:GABA-B1 OR I±:GABA-B1 OR D±:GluA2 OR I±:GluA2 OR D±:GluA1 OR I±:GluA1 OR D±:GluA3 OR I±:GluA3 OR D±:GluA4 OR

 I±:GluA4) OR Morphology:(Axons:DG:???? OR Axons:CA3:????? OR Axons:CA2:???? OR Axons:CA1:???? OR Axons:SUB:??? OR Axons:EC:?????? OR Soma:DG:???? OR Soma:CA3:????? OR Soma:CA2:???

? OR Soma:CA1:???? OR Soma:SUB:??? OR Soma:EC:?????? OR Dendrites:DG:???? OR Dendrites:CA3:????? OR Dendrites:CA2:???? OR Dendrites:CA1:???? OR Dendrites:SUB:??? OR Dendrites:EC:??

????)) AND Postsynaptic:(Markers:(D±:CB OR I±:CB OR D±:CR OR I±:CR OR D±:PV OR I±:PV OR D±:Mus2R OR I±:Mus2R OR D±:5HT-3 OR I±:5HT-3 OR D±:Gaba-a-alpha OR I±:Gaba-a-alpha OR D±:mGl

uR1a OR I±:mGluR1a OR D±:vGluT3 OR I±:vGluT3 OR D±:CCK OR I±:CCK OR D±:SOM OR I±:SOM OR D±:nNOS OR I±:nNOS OR D±:PPTA OR I±:PPTA OR D±:vGluT2 OR I±:vGluT2 OR D±:CGRP OR I±:CGRP OR

D±:mGluR2/3 OR I±:mGluR2/3 OR D±:mGluR5 OR I±:mGluR5 OR D±:Prox1 OR I±:Prox1 OR D±:GABAa \delta OR I±:GABAa \delta OR D±:MUS1R OR I±:MUS1R OR D±:Mus3R OR I±:Mus3R OR D±:Mus4R OR I±

:Mus4R OR D±:Cam OR I±:Cam OR D±:AMPAR 2/3 OR I±:AMPAR 2/3 OR D±:Disc1 OR I±:Disc1 OR D±:BONG OR I±:BONG OR D±:p-CREB OR I±:p-CREB OR D±:Neuropilin2 OR I±:Neuropilin2 OR D±:mGluR1

OR I±:mGluR1 OR D±:Caln OR I±:Caln OR D±:vGlut1 OR I±:vGlut1 OR D±:mGluR2 OR I±:mGluR2 OR D±:mGluR3 OR I±:mGluR3 OR D±:GABAa\alpha 2 OR I±:GABAa\alpha 2 OR D±:GABAa\alpha 3 OR I±:G

ABAa\alpha 3 OR D±:GABAa\alpha 4 OR I±:GABAa\alpha 4 OR D±:GABAa\alpha 5 OR I±:GABAa\alpha 5 OR D±:GABAa\alpha 6 OR I±:GABAa\alpha 6 OR D±:GABAa\beta 1 OR I±:GABAa\beta 1 OR D±:GAB

Aa\beta 2 OR I±:GABAa\beta 2 OR D±:GABAa\beta 3 OR I±:GABAa\beta 3 OR D±:GABAa\gamma 1 OR I±:GABAa\gamma 1 OR D±:GABAa\gamma 2 OR I±:GABAa\gamma 2 OR D±:mGluR5a OR I±:mGluR5a OR D±

:GlyT2 OR I±:GlyT2 OR D±:mGluR7a OR I±:mGluR7a OR D±:mGluR8a OR I±:mGluR8a OR D±:vAChT OR I±:vAChT OR D±:AChE OR I±:AChE OR D±:Kv3.1 OR I±:Kv3.1 OR D±:Cx36 OR I±:Cx36 OR D±:AR-beta

1 OR I±:AR-beta1 OR D±:AR-beta2 OR I±:AR-beta2 OR D±:TH OR I±:TH OR D±:mGluR4 OR I±:mGluR4 OR D±:CXCR4 OR I±:CXCR4 OR D±:GABA-B1 OR I±:GABA-B1 OR D±:GluA2 OR I±:GluA2 OR D±:GluA1 O

R I±:GluA1 OR D±:GluA3 OR I±:GluA3 OR D±:GluA4 OR I±:GluA4) OR Morphology:(Axons:DG:???? OR Axons:CA3:????? OR Axons:CA2:???? OR Axons:CA1:???? OR Axons:SUB:??? OR Axons:EC:??????

OR Soma:DG:???? OR Soma:CA3:????? OR Soma:CA2:???? OR Soma:CA1:???? OR Soma:SUB:??? OR Soma:EC:?????? OR Dendrites:DG:???? OR Dendrites:CA3:????? OR Dendrites:CA2:???? OR Dendrites:CA1:???? OR Dendrites:SUB:??? OR Dendrites:EC:??????)))

Requests for neurons return details for individual neurons while requests for connections return pairs of neurons connected through synapses.

Once a request is served, for either type, the following url is queried for neuron details:

[http://hippocampome.org/php/neuron_page.php?id={neuron_id}](http://hippocampome.org/php/neuron_page.php?id=%7bneuron_id%7d)

and a page scraping algorithm is applied (through the Beautiful Soup Python library) to extract the needed information.

**Elasticsearch storage format**

Data fetched from the source repositories are homogenized to the following dictionary format:

{

  “identifier”: {

   “source”: {

     “source_id”: value,

     “id”: value,

     “type”: value,

     “name”: value,

    “description”: value,

    “region”: value,

    “secondary_region”: value,

     “page_link”: value,

     “species”: value,

    “cell_type”: value,

    “download_link”: value,

}

}

}

In case some details are not available, the relative field is left empty, and the item metadata are not displayed.
